# Genomic insight into COVID-19 severity in MAFLD patients: a single-center prospective cohort study

**DOI:** 10.3389/fgene.2024.1460318

**Published:** 2024-09-04

**Authors:** Mykhailo Buchynskyi, Valentyn Oksenych, Iryna Kamyshna, Olena Budarna, Iryna Halabitska, Pavlo Petakh, Oleksandr Kamyshnyi

**Affiliations:** ^1^ Department of Microbiology, Virology, and Immunology, I. Horbachevsky Ternopil National Medical University, Ternopil, Ukraine; ^2^ Broegelmann Research Laboratory, Department of Clinical Science, University of Bergen, Bergen, Norway; ^3^ Department of Medical Rehabilitation, I. Horbachevsky Ternopil National Medical University, Ternopil, Ukraine; ^4^ Department of Neurology, I. Horbachevsky Ternopil National Medical University, Ternopil, Ukraine; ^5^ Department of Therapy and Family Medicine, I. Horbachevsky Ternopil National Medical University, Ternopil, Ukraine; ^6^ Department of Biochemistry and Pharmacology, Uzhhorod National University, Uzhhorod, Ukraine

**Keywords:** MAFLD, COVID-19, ACE2, IFNAR2, SNP

## Abstract

This study investigated the influence of single nucleotide polymorphisms (SNPs) in genes associated with the interferon pathway (IFNAR2 rs2236757), antiviral response (OAS1 rs10774671, OAS3 rs10735079), and viral entry (ACE2 rs2074192) on COVID-19 severity and their association with nonalcoholic fatty liver disease (MAFLD). We did not observe a significant association between the investigated SNPs and COVID-19 severity. While the IFNAR2 rs2236757 A allele was correlated with higher creatinine levels upon admission and the G allele was correlated with lower band neutrophils upon discharge, these findings require further investigation. The distribution of OAS gene polymorphisms (rs10774671 and rs10735079) did not differ between MAFLD patients and non-MAFLD patients. Our study population’s distribution of ACE2 rs2074192 genotypes and alleles differed from that of the European reference population. Overall, our findings suggest that these specific SNPs may not be major contributors to COVID-19 severity in our patient population, highlighting the potential role of other genetic factors and environmental influences.

## 1 Introduction

Coronavirus disease 2019 (COVID-19) is a respiratory illness resulting from infection with severe acute respiratory syndrome coronavirus 2 (SARS-CoV-2). This highly pathogenic coronavirus is highly contagious and has the potential to cause severe respiratory complications.

The vast majority of COVID-19 patients (approximately 85%) exhibit mild symptoms. However, a subset of infected individuals (roughly 5%) progress to severe illness, characterized by acute respiratory distress syndrome (ARDS) and damage to multiple organs (Pfortmueller et al., 2021). The emergence of COVID-19 has resulted in a significant global pandemic, with substantial morbidity and mortality. As of this manuscript’s writing, the worldwide death toll attributable to COVID-19 has surpassed 7 million (WHO, 2020). A critical distinction between SARS-CoV-2 and earlier coronavirus strains lies in the heightened virulence of SARS-CoV-2, which translates to more severe clinical presentations (Triggle et al., 2021).

A growing body of evidence indicates that host-related factors significantly influence infection outcomes. Specifically, advanced age (Rapp et al., 2021; Zhang et al., 2021; Ebinge et al., 2020; Williamson et al., 2020), male sex (Rapp et al., 2021; Ebinge et al., 2020; Williamson et al., 2020), the presence of hypertension (Li et al., 2020; Kamyshnyi et al., 2020), cardiovascular disease (Williamson et al., 2020) obesity (Rapp et al., 2021; Zhang et al., 2021; Gao et al., 2020; Valentino et al., 2023), and type 2 diabetes (Williamson et al., 2020; You et al., 2020; Dennis et al., 2021; Petakh et al., 2022; Kamyshnyi et al., 2021) have been consistently associated with increased infection susceptibility or severity.

Although these factors have been identified as risk indicators for severe disease manifestations, they alone are insufficient to account for the observed heterogeneity in COVID-19 severity across individuals.

Recent research suggests that tiny variations in our genes, called SNPs (single nucleotide polymorphisms), might affect how likely we are to develop COVID-19 and how severe it could be (Buchynskyi et al., 2023a; Fricke-Galindo and Falfán-Valencia, 2021; Pairo-Castineira et al., 2020; Negi et al., 2023). These SNPs are likely to exert their effects through various biological pathways implicated in the disease process. The intricate interplay between an individual’s genetic architecture, characterized by a unique constellation of single nucleotide polymorphisms (SNPs), is hypothesized to significantly influence the pathogenesis and clinical course of COVID-19.

Coronaviruses, including SARS-CoV-2, utilize spike (S) glycoproteins to bind to host cell receptors, facilitating viral entry and membrane fusion (Li, 2016). Angiotensin-converting enzyme 2 (ACE2), expressed on the cell surface, serves as the primary receptor for SARS-CoV-2 attachment (Harrison et al., 2020). Higher levels of ACE2 expression translate to increased numbers of viral binding sites on host cells, potentially rendering individuals more susceptible to infection (Li, 2013). Notably, ACE2 rs2074192 has been associated with an increased risk of arterial hypertension in obese males (Cui et al., 2021). Additionally, this polymorphism has been implicated in the pathogenesis of both type 2 diabetes mellitus (T2DM) and cardiovascular disease (Liu et al., 2018). A variation in the ACE2 gene, called rs2074192, might affect how effectively the instructions for building the ACE2 protein are followed. This variation is found in a noncoding region, but it could still influence how the ACE2 gene is processed. This disruption could change how strongly the virus that causes COVID-19 binds to human cells (Pouladi and Abdolahi, 2021). A study by Sienko et al. supported this idea, finding a link between the rs2074192 variation and the severity of COVID-19 in adults (Sienko et al., 2022).

Innate immunity represents a critical line of defense against SARS-CoV-2 infection. Interferons (IFNs), which display both antiproliferative and immunoregulatory properties, are essential mediators of the innate immune response against viral infections (Hammad et al., 2024; Kamyshnyi et al., 2023; Buchynskyi et al., 2023b). Polymorphisms within IFN genes or their receptors have been implicated in heightened susceptibility to COVID-19 and more severe clinical outcomes (Pairo-Castineira et al., 2020; Fricke-Galindo et al., 2022; Dieter et al., 2022). A large genetic study (GWAS) by Pairo-Castineira et al. (2020) revealed a strong connection between a specific variation (rs2236757) in the IFNAR2 gene and worsening of COVID-19 symptoms (Pairo-Castineira et al., 2020). This link between IFNAR2 variations and severity was further supported by Fricke-Galindo et al. (2022), who discovered that several variations were associated with a greater risk of death in COVID-19 patients. Higher levels of soluble IFNAR2 (sIFNAR2) were observed in survivors, suggesting that enhanced antiviral activity was facilitated by sIFNAR2 stability (Fricke-Galindo et al., 2022).

Another critical genetic mechanism involves three specific genes, OAS1, OAS2, and OAS3, which encode enzymes called OAS enzymes that have antiviral properties (Kozak et al., 2023). These enzymes work by activating another molecule, RNase L (the latent form of ribonuclease L), which helps fight viruses (Sadler and Williams, 2008; Kerr et al., 1977). The RNase L pathway plays a significant role in the host immune response against SARS-CoV-2 infection (Pairo-Castineira et al., 2020).

A recent study by Banday et al. (2022) showed a strong connection between a variation (rs10774671) in the OAS1 gene and how much of the OAS1 protein is produced (Banday et al., 2022). This OAS1 protein is an important weapon used by the body to fight viruses, including SARS-CoV-2. Their study suggested that this polymorphism’s functional impact on OAS1 protein abundance contributes to its association with COVID-19 hospitalization outcomes (Banday et al., 2022). In another study examining genes involved in the antiviral response, Pairo-Castineira et al. (2020) reported a connection between a variation (rs10735079) in the OAS3 gene and an increased risk of severe COVID-19 illness (Pairo-Castineira et al., 2020). This suggests that variations in these genes, such as OAS1 and OAS3, might influence our susceptibility to severe COVID-19.

Deciphering the genetic basis of COVID-19 severity is a formidable task due to the remarkable complexity of human genetics. Numerous genes interact in intricate pathways, making it challenging to isolate the precise genetic factors at play.

Metabolic disorders exhibit a substantial prevalence among individuals succumbing to COVID-19, constituting up to 50% of fatalities (Steenblock et al., 2021). Patients with metabolic conditions such as obesity, hypertension, diabetes, and non-alcoholic fatty liver disease are at an increased risk of severe COVID-19. Moreover, SARS-CoV-2 infection can precipitate the onset of diabetes or exacerbate pre-existing metabolic disturbances (Kamyshnyi et al., 2021; Negi et al., 2023).

Metabolic-associated fatty liver disease (MAFLD) represents the most prevalent cause of chronic liver disease globally, affecting a substantial proportion of the population (Younossi et al., 2016; Powell et al., 2021). This spectrum of liver conditions encompasses a range from simple steatosis (fat accumulation) to more severe presentations characterized by inflammation and potential progression to cirrhosis (Eslam et al., 2020a; Eslam et al., 2020b). Notably, individuals diagnosed with MAFLD exhibit elevated levels of interleukin-6 (IL-6) (Gao et al., 2021). IL-6 is a critical cytokine implicated in the inflammatory cytokine storm observed in patients with severe COVID-19 (Dietz et al., 2021; Inciardi et al., 2020; Nowroozi et al., 2023). This finding suggested that MAFLD may exacerbate COVID-19 outcomes by further amplifying this inflammatory response.

Chronic inflammation, a well-established contributor to the pathogenesis of fatty liver disease, is orchestrated by hepatic and adipose tissue macrophages through the secretion of cytokines and adipokines (Kazankov et al., 2019). Notably, MAFLD appears to exacerbate the overreaction of the immune system observed in patients with severe COVID-19. This occurs because MAFLD is linked to the release of a flood of inflammatory molecules, including interleukin-6 (IL-6), which contributes to the cytokine storm (Gao et al., 2021). Recent studies have highlighted the potential importance of interferon regulatory factors (IRFs) in MAFLD, with these molecules playing a critical role in the induction of interferon (IFN) transcription (Møhlenberg et al., 2019).

However, it is unclear whether MAFLD is merely a risk factor for more severe COVID-19 outcomes or directly contributes to COVID-19 pathogenesis (Buchynskyi et al., 2023c; Buchynskyi et al., 2024).

## 2 Materials and methods

### 2.1 Research approach and participants

This prospective cohort study was undertaken at the I. Horbachevsky Ternopil National Medical University (TNMU) in Ternopil, Ukraine. The study population included individuals of European ancestry (of Ukrainian ethnicity) and ranged in age from 23 to 86 years.

The study enrolled a total of 72 adult participants who tested positive for SARS-CoV-2 via nasopharyngeal swab samples analysed using real-time polymerase chain reaction (RT‒PCR) (Dieter et al., 2022). All participants were admitted to the hospital between October 2022 and May 2023. A control group of 24 patients without COVID-19 and MAFLD was also included.

The inclusion criterion was adults who were diagnosed with COVID-19 and needed to be hospitalized. The severity of their illness was categorized based on guidelines set by the National Institutes of Health (NIH) as moderate, severe, or critical (Clinical Spectrum, 2023); control group patients had similar baseline characteristics and confirmed absence of COVID-19.

The exclusion criteria were as follows: death within 48 h of admission to the hospital, preenrollment use of corticosteroids, palliative care, serious bacterial infection upon admission, cirrhosis or chronic liver disease, alcohol dependence, weakened immune systems, and HIV infection.

All participants were checked for signs of metabolic syndrome. The diagnosis of MAFLD was then established based on the current criteria. The identification of steatosis usually consists of the use of various modalities (imaging, blood biomarkers, or histology) in conjunction with the following criteria: T2DM, overweight/obesity or demonstrable evidence of metabolic abnormalities (Fouad et al., 2020; Méndez-Sánchez et al., 2022). To assess the presence of hepatic steatosis, we employed the hepatic steatosis index (HSI). This scoring system considers factors such as body mass index, liver enzymes, and the presence of diabetes to estimate the likelihood of fat accumulation in the liver (Cui et al., 2021).

Following the National Institutes of Health (NIH) guidelines (Clinical Spectrum, 2023), participants were stratified into three subgroups based on COVID-19 severity: moderate – characterized by bilateral pneumonia with oxygen saturation (SpO_2_) ≥ 94% on room air; severe – defined by the presence of dyspnea (difficulty breathing) and/or tachypnea (respiratory rate > 24 breaths/minute) and/or SpO_2_ < 94%; critical – requiring intensive care unit (ICU) admission, fulfilling the criteria for acute respiratory distress syndrome (ARDS), or necessitating advanced respiratory support using a high-flow nasal cannula (HFNC), non-invasive ventilation, or invasive mechanical ventilation.

Following enrollment and application of the inclusion/exclusion criteria, the final study sample comprised 33 participants who were diagnosed with COVID-1 and MAFLD, 39 participants who were diagnosed with COVID-19 without MAFLD, and a control group of 24 individuals. All participants were naïve to prior research participation and provided written informed consent. The study was approved by a special committee at I. Horbachevsky Ternopil National Medical University that oversees research ethics (protocol no. 74).

### 2.2 Laboratory and clinical data

As part of the standard diagnostic workup, a comprehensive laboratory analysis was conducted. These parameters included hematological indices (white blood cell count with differential, erythrocyte sedimentation rate, hematocrit, and platelet count), coagulation parameters (international normalized ratio, prothrombin time, activated partial thromboplastin time, and fibrinogen), liver function tests (total bilirubin, alanine aminotransferase, and aspartate aminotransferase), renal function (creatinine), markers of cholestasis (gamma-glutamyl transferase), and inflammatory markers (C-reactive protein). Additionally, blood glucose levels were measured. Body mass index (BMI) was recorded for all participants.

Genomic DNA isolation: Peripheral blood leukocytes were used to isolate genomic DNA for subsequent genotyping analysis. This procedure employed a commercially available kit (Thermo Scientific™ GeneJET™ Whole Blood Genomic DNA Purification Mini Kit). Briefly, 200 µL of whole blood from each participant was digested with proteinase K, followed by the addition of lysis buffer. The subsequent steps involved washing and elution of the purified DNA.

Genotyping analysis: Real-time polymerase chain reaction (RT‒PCR) was used to analyse polymorphisms in four genes: ACE2 (rs2074192), IFNAR2 (rs2236757), OAS1 (rs10774671), and OAS3 (rs10735079). The CFX96™ Real-Time PCR Detection System (Bio-Rad Laboratories, Inc., United States) was used for this purpose. Specific TaqMan™ SNP genotyping assays were utilized for each targeted SNP. Amplification of the DNA was achieved using TaqMan™ Universal Master Mix II.

Genotyping assays were conducted on all specimens utilizing TaqMan^®^ probes and TaqMan^®^ Genotyping Master Mix (4371355) in conjunction with the CFX96™ Real-Time PCR Detection System. The PCR protocol strictly adhered to the manufacturer’s instructions (Applied Biosystems, United States). The TaqMan^®^ Genotyping Master Mix included AmpliTaq Gold^®^ DNA polymerase, dNTPs, ROX™ reference dye, and optimized reaction buffers. TaqMan^®^ probes are allele-specific oligonucleotides with reporter dyes (VIC^®^ for Allele 1 and 6-FAM™ for Allele 2) attached to the 5′ end and a nonfluorescent quencher (NFQ) at the 3′ end. Genomic DNA (10 μL) was amplified in a reaction mixture containing primers, probes, Master Mix, and the target DNA. Allele discrimination based on relative fluorescence units (RFUs) was employed for genotyping using CFX-Manager™ software.

The PCR cycling conditions were as follows: initial denaturation at 95°C for 10 min; amplification cycles (49 cycles); denaturation at 95°C for 15 s; annealing at 60°C for 1:10 min; and a final melting curve analysis at 95°C. Genotype determination was performed based on melting curve analysis using CFX96™ Real-Time PCR Basic Software (Bio-Rad Laboratories, Inc., United States).

### 2.3 Statistical analysis

In this study, a comprehensive statistical analysis was performed to evaluate the collected data. Demographic information, clinical characteristics, and laboratory parameters were meticulously assessed and presented using descriptive statistics. To describe the data, we reported how often things occurred (frequencies) and the middle values (medians) along with the spread of the data (interquartile ranges).

To investigate groupwise differences in categorical variables, appropriate statistical tests were chosen based on sample size and table dimensions. Frequency tables with 2 × 3 dimensions were analysed using the chi-square test (χ^2^), while 2 × 2 tables were analysed with the two-tailed Fisher exact test. The significance level (*p*-value) was calculated for each test. Genotype frequencies were assessed for conformance to Hardy‒Weinberg equilibrium (HWE) to ensure that the study population reflected the expected distribution of alleles in the target population (*p* > 0.05 in the chi-square test).

To identify key predictors of COVID-19 severity within the study population, logistic regression analysis was performed. Statistical significance was set at a *p*-value of less than 0.05. Furthermore, GeneMANIA network data were utilized to evaluate potential interactions between the investigated genes (GENEMANIA, 2024). *A priori* sample size calculations and *post hoc* power analysis were performed using “G*Power 3.1.9.7.”

For comparisons between two independent groups, the nonparametric Mann–Whitney U test was employed. In the case of three or more groups, statistical comparisons were performed using the Kruskal–Wallis test, a nonparametric alternative to one-way ANOVA. Dunn’s multiple comparisons test was subsequently employed for pairwise comparisons between groups. All the statistical tests were two-tailed, with a *p*-value less than 0.05 indicating statistical significance. The point-biserial correlation coefficient was utilized to assess the relationship between binary and continuous data within a correlation matrix. To evaluate the performance of the binary logistic regression model, ROC analysis was conducted. The strength of association between variables was expressed as an odds ratio (OR) along with its corresponding 95% confidence interval (CI). Statistical software programs, including GraphPad Prism (version 8.4.3), IBM SPSS Statistics (version 25), and Jamovi (version 2.4.11), were used for all the statistical analyses.

## 3 Results

### 3.1 Sample characteristics

A total of 96 patients were included in the study and categorized into three groups: COVID-19 patients with MAFLD (n = 33, 64% male, median age 66 years, interquartile range [IQR] 50–72 years), COVID-19 patients without MAFLD (n = 39, 56% male, median age 65 years, IQR 41–72 years), and controls (n = 39, 71% male, median age 50 years, IQR 38.75–58.5 years) (Table 1).

**TABLE 1:** Baseline patient characteristics.

	COVID-19 with MAFLD (n = 33)	COVID-19 without MAFLD (n = 39)	Control (n = 24)	*p*-value^a^
Age (years), IQR^b^	66 (50–72)	65 (41–72)	50 (38.75–58.5)	0.011
Male, No. (%)	21 (64%)	22 (56%)	17 (71%)	0.510
BMI^c^, kg/m^2^	30.8 (28.42–33.55)	24 (22.4–25.35)	24.1 (22.95–25.75)	<0.001
Comorbidities				
Diabetes mellitus	14 (42%)	2 (5%)	1 (4%)	<0.001
Arterial hypertension	25 (76%)	18 (46%)	5 (21%)	<0.001
Coronary heart disease	14 (42%)	13 (33%)	1 (4%)	<0.001
COPD^d^	3 (9%)	1 (3%)	0	0.307

^a^ Chi-squared, Kruskal–Wallis test with Dunn’s multiple comparisons test.

^b^ Data are reported as medians and interquartile ranges (IQR).

^c^ BMI, body mass index.

^d^ COPD, chronic obstructive pulmonary disease.

Patients with COVID-19 and MAFLD were significantly older than those in the control group were (median age 66 years vs. 50 years, *p* = 0.011). The COVID-19 patients in the MAFLD group had a significantly greater BMI than did those in both the non-MAFLD group (30.8 vs. 24.0, *p* < 0.001) and the control group (30.8 vs. 24.1, *p* < 0.001). The prevalence of type 2 diabetes mellitus (T2DM), arterial hypertension, and coronary heart disease was significantly greater in the COVID-19 with MAFLD group than in the other two groups (all *p* values < 0.001).

The sex distribution (χ^2^ = 1.346, *p* = 0.510) and presence of chronic obstructive pulmonary disease (COPD) (χ^2^ = 2.364, *p* = 0.510) did not significantly differ between the groups.

The distribution of genotypes for the ACE2 rs2074192, IFNAR2 rs2236757, OAS1 rs10774671, and OAS3 rs10735079 polymorphisms conformed to HWE in both the COVID-19 and control groups (*p* > 0.05). Detailed data on genotype frequencies are presented in Table 2.

**TABLE 2:** Tests for Hardy‒Weinberg equilibrium were performed for IFNAR2 rs2236757, ACE2 rs2074192, OAS1 rs10774671 and OAS3 rs10735079.

Group	Genotype	ACE2 rs2074192		Genotype	IFNAR2 rs2236757		OAS1 rs10774671		OAS3 rs10735079	
		Expected	Expected		Expected	Observed	Expected	Observed	Expected	Observed
COVID-19 patients	CC	41.25	40	AA	8	7	30.68	32	29.39	30
	CT	26.29	29	AG	32	34	32.64	30	33.22	32
	TT	4.25	3	GG	32	31	8.68	10	9.39	10
		χ^2^ = 0.645; *p* = 0.422			χ^2^ = 0.2813; *p* = 0.596		χ^2^ = 0.471; *p* = 0.493		χ^2^ = 0.097; *p* = 0.755	
Control group	CC	12.76	13	AA	2.34	2	14.26	14	13.5	13
	CT	9.48	9	AG	10.31	11	8.48	9	9	10
	TT	1.76	2	GG	11.34	11	1.26	1	1.5	1
		χ^2^ = 0.061; *p* = 0.804			χ^2^ = 0.107; *p* = 0.744		χ^2^ = 0.091; *p* = 0.763		χ^2^ = 0.296; *p* = 0.586	

### 3.2 Genotype and allele distribution in the studied groups

**FIGURE 1:**
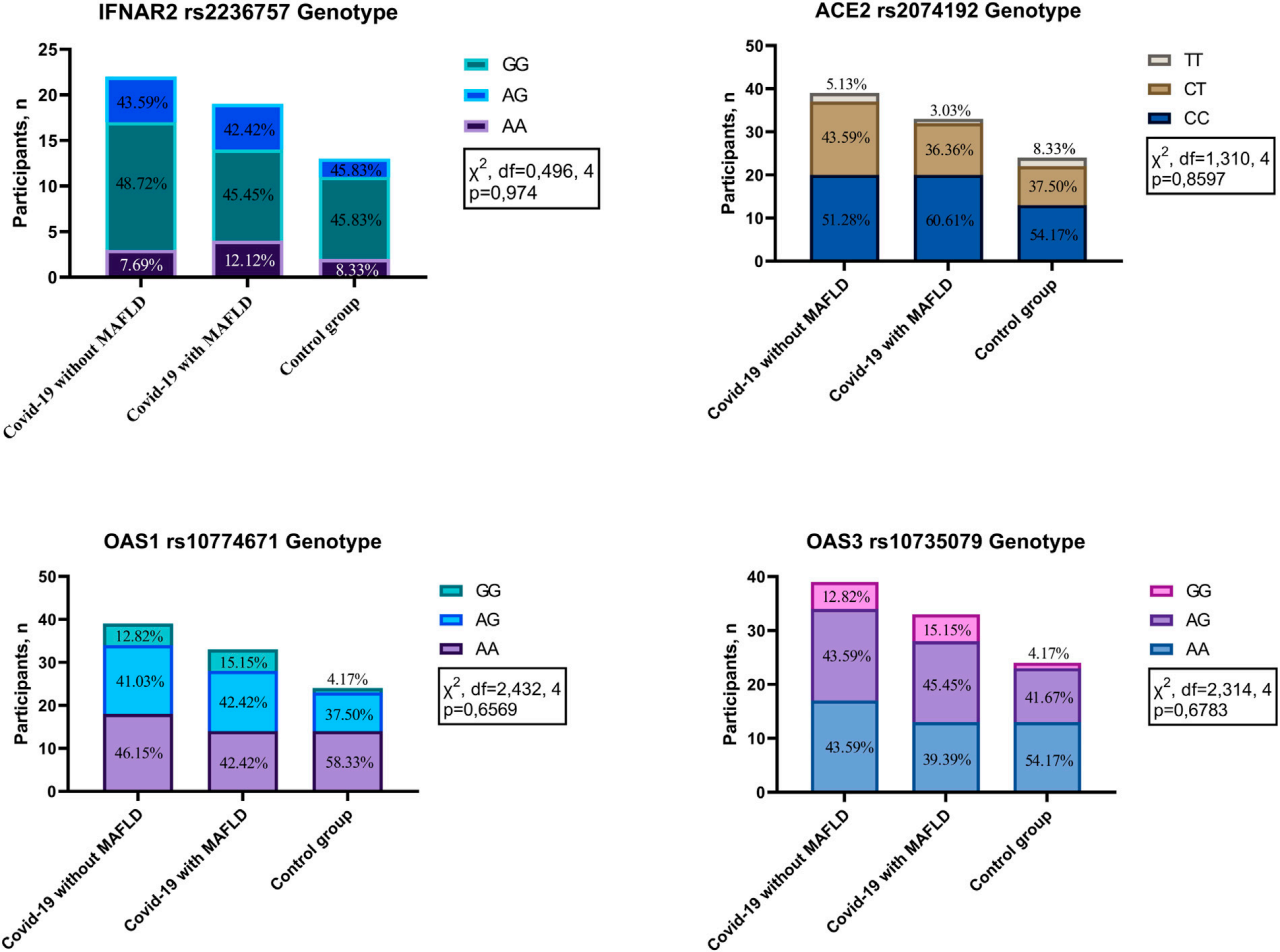
Genotype distribution in patients with COVID-19 with MAFLD vs. without MAFLD vs. controls. IFNAR2—interferon alpha and beta receptor subunit; ACE2—angiotensin converting enzyme 2; OAS1—2′-5′-oligoadenylate synthetase 1; OAS3—2′-5′-oligoadenylate synthetase 3.

No statistically significant differences in genotype frequencies were detected among the investigated groups (COVID-19 patients with MAFLD, COVID-19 patients without MAFLD, and controls) for the four polymorphisms analysed: IFNAR2 rs2236757 (χ^2^ = 0.496, df = 4, *p* = 0.974), ACE2 rs2074192 (χ^2^ = 1.310, df = 4, *p* = 0.8597), OAS1 rs10774671 (χ^2^ = 2.432, df = 4, *p* = 0.6569), and OAS3 rs10735079 (χ^2^ = 2.314, df = 4, *p* = 0.6783) (Figure 1). These findings suggest that genetic variations within these genes are not associated with MAFLD or COVID-19 susceptibility in the studied population.

Analysis revealed statistically significant differences in the genotype and allele frequencies of the ACE2 rs2074192 polymorphism between the study groups (COVID-19 patients with MAFLD, COVID-19 patients without MAFLD, and controls) and the European reference population (χ^2^ = 16.86, df = 4, *p* < 0.001 for genotype; Fisher’s exact test, *p* < 0.001 for allele). Notably, a difference in allele frequency was also observed when comparing the control group to the European population. In contrast, no significant differences in genotype or allele frequencies were observed for the other investigated polymorphisms (IFNAR2 rs2236757, OAS1 rs10774671, and OAS3 rs10735079) when comparing the study groups with the European population (Table 3).

**TABLE 3:** Analysis of allele frequencies in SARS-CoV-2 patients compared to a European population.

Genotype/Allele frequency								
Gene			Patients with COVID-19, n (%)	Control group, n (%)	European population, n (%)	*p*-value^a^ COVID-19-control	*p*-value^a^ COVID-19-EUR	*p*-value^a^ Control-EUR
IFNAR2 rs2236757	Genotype	AA	7 (10)	2 (8)	105 (10)	χ^2^ = 0,0762 *p* = 0.963	χ^2^ = 2.411 *p* = 0.299	χ^2^ = 0.649 *p* = 0.722
		AG	34 (47)	11 (46)	190 (38)			
		GG	31 (43)	11 (46)	260 (52)			
	Allele	A	48 (33)	15 (31)	296 (29)	Fisher’s exact test *p* = 0.860	Fisher’s exact test *p* = 0.332	Fisher’s exact test *p* = 0.750
		G	96 (66)	33 (69)	710 (71)			
ACE2 rs2074192	Genotype	CC	40 (56)	13 (54)	126 (33)	χ^2^ = 0.641 *p* = 0.726	χ^2^ = 16.86 *p* < 0.001	χ^2^ = 4.831 *p* = 0.089
		CT	29 (40)	9 (38)	188 (49)			
		TT	3 (4)	2 (8)	69 (18)			
	Allele	C	109 (76)	35 (73)	440 (57)	Fisher’s exact test *p* = 0.703	Fisher’s exact test *p* < 0.001	Fisher’s exact test *p* = 0.035
		T	35 (24)	13 (27)	326 (43)			
OAS1 rs10774671	Genotype	AA	32 (44)	14 (54)	209 (42)	χ^2^ = 2,145 *p* = 0.342	χ^2^ = 0.648 *p* = 0.723	χ^2^ = 3.121 *p* = 0.210
		AG	30 (42)	9 (42)	234 (46)			
	
		GG	10 (14)	1 (4)	60 (12)			
	Allele	A	94 (65)	37 (77)	652 (64)	Fisher’s exact test *p* = 0.153	Fisher’s exact test *p* = 1.000	Fisher’s exact test *p* = 0.089
		G	50 (35)	11 (23)	354 (36)			
OAS3 rs10735079	Genotype	AA	30 (42)	13 (59)	201 (40)	χ^2^ = 2,286 *p* = 0.319	χ^2^ = 0.223 *p* = 0.895	χ^2^ = 2.654 *p* = 0.265
		AG	32 (44)	10 (37)	238 (47)			
		GG	10 (14)	1 (4)	64 (13)			
	Allele	A	92 (64)	36 (75)	640 (65)	Fisher’s exact test *p* = 0.215	Fisher’s exact test *p* = 1.000	Fisher’s exact test *p* = 0.124
		G	52 (36)	12 (25)	366 (35)			

^a^ Fisher exact and chi-squared tests, as appropriate.

### 3.3 Risk alleles and genotype in COVID-19 patients

Analysis of the investigated polymorphisms (details provided in Table 4) revealed no statistically significant differences in allele frequencies among the three groups: COVID-19 with moderate severity, COVID-19 with severe/critical illness, and the control group. These findings suggest that the specific genetic variations analysed do not appear to be associated with the severity of disease in COVID-19 patients.

**TABLE 4:** Allele influence on COVID-19 severity.

Gene	Allele	COVID-19 severity			^a^χ^2^; *p*-value	^b^ *p*-value (moderate to severe/critical COVID-19)	^c^OR (CI for OR)
		Moderate n = 42	Severe/critical n = 30	Control, n = 24			
IFNAR2 rs2236757	A	27	21	15	χ^2^ = 0.200; *p* = 0.905	*p* = 0.724	0.880 (0.4368–1.812)
	G	57	39	33			
ACE2 rs2074192	C	67	42	35	χ^2^ = 1.927; *p* = 0.382	*p* = 0.237	1.689 (0.7781–3.717)
	T	17	18	13			
OAS3 rs10735079	A	52	40	36	χ^2^ = 2.357; *p* = 0.308	*p* = 0.601	0.812 (0.3955–1.599)
	G	32	20	12			
OAS1 rs10774671	A	53	41	37	χ^2^ = 2.758; *p* = 0.252	*p* = 0.595	0.7923 (0.4056–1.579)
	G	31	19	11			

^a^ Chi-squared test and.

^b^ Fisher exact test, as appropriate.

^c^ Odds ratio.

Analysis of laboratory parameters at admission revealed associations between specific genotypes and certain markers (detailed data are presented in Table 5). Patients carrying the A allele of IFNAR2 rs2236757 exhibited significantly greater creatinine levels than did those without the A allele (88.5 mmol/L vs. 50 mmol/L, IQRs: 50–72 vs. 38.75–58.5 mmol/L, *p* = 0.021).

**TABLE 5:** Associations between genotype and laboratory findings at hospital admission in COVID-19 patients.

IFNAR2 rs2236757						
	Allele A (n = 41)	No Allele A (n = 31)	*p*-value^a^	Allele G (n = 65)	No Allele G (n = 7)	*p*-value^a^
Creatinine, mmol/L	103 (88.5–121)	90 (71–102)	*p* = 0.021	96 (81.5–110)	104 (80–120)	*p* = 0.481

ACE2 rs2074192						
	Allele С (n = 69)	No Allele С (n = 3)	*p*-value^a^	Allele T (n = 32)	No Allele T (n = 40)	*p*-value^a^
Band neutrophils, %	9 (6–13.5)	3 (2–7)	*p* = 0.046	7 (5.25–12)	9 (6–14.8)	*p* = 0.625
Total bilirubin, mmol/L	12.4 (10.8–14.9)	21.9 (20.5–107)	*p* = 0.004	13.8 (10.8–18.9)	12.2 (10.7–14.2)	*p* = 0.162
Eosinophils, %	1 (0–1.5)	1 (0–3)	*p* = 0.915	1 (0–1)	1 (0–2)	*p* = 0.036

OAS1 rs10774671						
	Allele A (n = 62)	No Allele A (n = 10)	*p*-value^a^	Allele G (n = 40)	No Allele G (n = 30)	*p*-value^a^
Fibrinogen, g/L	3.77 (3.11–5.16)	3.85 (3.55–5.16)	*p* = 0.598	3.99 (3.55–5.49)	3.52 (2.88–4.83)	*p* = 0.033
General protein, g/L	68.6 (62.2–73.7)	68 (63.2–72.7)	*p* = 0.907	70.4 (64.5–75.1)	66.9 (60.9–70.4)	*p* = 0.032

OAS3 rs10735079						
	Allele A (n = 62)	No Allele A (n = 10)	*p*-value^a^	Allele G (n = 42)	No Allele G (n = 30)	*p*-value^a^
General protein, g/L	68.6 (62.2–73.7)	68 (63.2–72.7)	*p* = 0.907	70.4 (64.8–74.8)	66.2 (60.2–70.7)	*p* = 0.011

^a^ Mann–Whitney U test, as appropriate.

For ACE2 rs2074192, the presence of the C allele was associated with both lower band neutrophil levels (9 mmol/L vs. 3 mmol/L, IQRs: 6–13.5 vs. 2–7 mmol/L, *p* = 0.046) and higher total bilirubin levels (12.4 mmol/L vs. 21.9 mmol/L, IQRs: 10.8–14.9 vs. 20.5–107 mmol/L, *p* = 0.004). Additionally, the G allele was linked to lower eosinophil levels (1% vs. 1%, IQRs: 0%–1% vs. 0%–2%, *p* = 0.036).

For OAS1 rs10774671, the G allele was associated with elevated fibrinogen (3.99 g/L vs. 3.52 g/L, IQRs: 3.55–5.49 vs. 2.88–4.83 g/L, *p* = 0.033) and total protein levels (70.4 g/L vs. 66.9 g/L, IQRs: 64.5–75.1 vs. 60.9–70.4 g/L, *p* = 0.032).

Last, the G allele of OAS3 rs10735079 was linked to higher total protein levels (70.4 g/L vs. 66.2 g/L, IQRs: 64.8–74.8 vs. 60.2–70.7 g/L, *p* = 0.011).

Further analysis of laboratory parameters upon hospital discharge revealed additional associations between specific genotypes and certain markers (detailed data in Table 6). Patients carrying the C allele of ACE2 rs2074192 exhibited significantly lower international normalized ratios (INRs) (1.01 vs. 1.09, IQRs: 0.92–1.07 vs. 1.07–1.17, *p* = 0.046) and prothrombin times (PTs) (12.6 s vs. 13.6 s, IQRs: 11.85–13.4 vs. 13.4–14.4 s, *p* = 0.045) than did those without the C allele.

**TABLE 6:** Associations between genotype and laboratory findings at hospital discharge in COVID-19 patients.

ACE2 rs2074192						
	Allele С (n = 69)	No Allele С (n = 3)	*p*-value^a^	Allele T (n = 32)	No Allele T (n = 40)	*p*-value^a^
Leukocytes, 10^9^/L	8.93 (6.15–11.3)	6.99 (5.17–7.37)	*p* = 0.299	7.55 (5.35–9.24)	9.34 (6.39–12.46)	*p* = 0.051
INR*, n	1.01 (0.92–1.07)	1.09 (1.07–1.17)	*p* = 0.046	1.01 (0.92–1.07)	1.05 (0.95–1.09)	*p* = 0.281
PT*, sec	12.6 (11.85–13.4)	13.6 (13.4–14.4)	*p* = 0.045	12.55 (11.7–13.4)	12.85 (12.2–13.4)	*p* = 0.571
QPT*, %	96.2 (82.2–104.7)	79.3 (71.6–84.3)	*p* = 0.053	98.65 (83.18–102.1)	91.15 (81.4–106.3)	*p* = 0.869

OAS1 rs10774671						
	Allele A (n = 62)	No Allele A (n = 10)	*p*-value^a^	Allele G (n = 40)	No Allele G (n = 32)	*p*-value^a^
Hematocrit, %	36 (31.34–42.18)	38.36 (34.96–44.2)	*p* = 0.179	37.91 (32.95–42.99)	34.65 (30.65–39.55)	*p* = 0.044
General protein, g/L	64.55 (60.88–70.93)	67.25 (61.58–72.23)	*p* = 0.444	66.6 (62.45–72.15)	63.4 (58.35–68.78)	*p* = 0.011

OAS3 rs10735079						
	Allele A (n = 62)	No Allele A (n = 10)	*p*-value^a^	Allele G (n = 42)	No Allele G (n = 30)	*p*-value^a^
Monocytes, %	5 (3–7.25)	5 (2–9.25)	*p* = 0.725	5 (4–8)	4 (2–6.5)	*p* = 0.049
Hematocrit, %	36 (31.34–42.18)	38.36 (34.96–44.20)	*p* = 0.179	37.91 (32.77–43)	33.84 (30.35–38.12)	*p* = 0.024
General protein, g/L	64.55 (60.88–70.93)	67.25 (61.58–72.23)	*p* = 0.444	66.6 (61.98–72.23)	63.5 (58.08–68.73)	*p* = 0.016

Abbreviations: INR, international normalized ratio; PT, prothrombin time; QPT, quick prothrombin time.

^a^ Mann–Whitney U test, as appropriate.

The presence of the G allele of OAS1 rs10774671 was associated with both elevated hematocrit (37.91% vs. 34.65%, IQRs: 32.95%–42.99% vs. 30.65%–39.55%, *p* = 0.044) and increased total protein levels (66.6 g/L vs. 63.4 g/L, IQRs: 62.45–72.15 vs. 58.35–68.78 g/L, *p* = 0.011) upon discharge.

Similarly, the G allele of OAS3 rs10735079 was linked to increased monocyte count (5% vs. 4%, IQRs: 4%–8% vs. 2%–6.5%, *p* = 0.049), haematocrit (37.91% vs. 33.94%, IQRs: 32.77%–43% vs. 30.35%–38.12%, *p* = 0.024), and total protein levels (66.6 g/L vs. 63.5 g/L, IQRs: 61.98–72.23 vs. 58.08–68.73 g/L, *p* = 0.016) at discharge.

Upon admission, patients with the ACE2 rs2074192 CC genotype exhibited significantly lower total bilirubin levels than did those with the TT genotype (median 12.2 mmol/L, interquartile range [IQR] 10.7–14.2 mmol/L vs. median 21.9 mmol/L, IQR 20.5–107 mmol/L, *p* = 0.024). Additionally, the presence of the OAS3 rs10735079 AA genotype was associated with higher general protein levels than the presence of the OAS3 AG genotype (median 66.2 g/L, IQR 60.2–70.7 g/L vs. median 70.4 g/L, IQR 65.6–75.1 g/L, *p* = 0.019).

**FIGURE 2:**
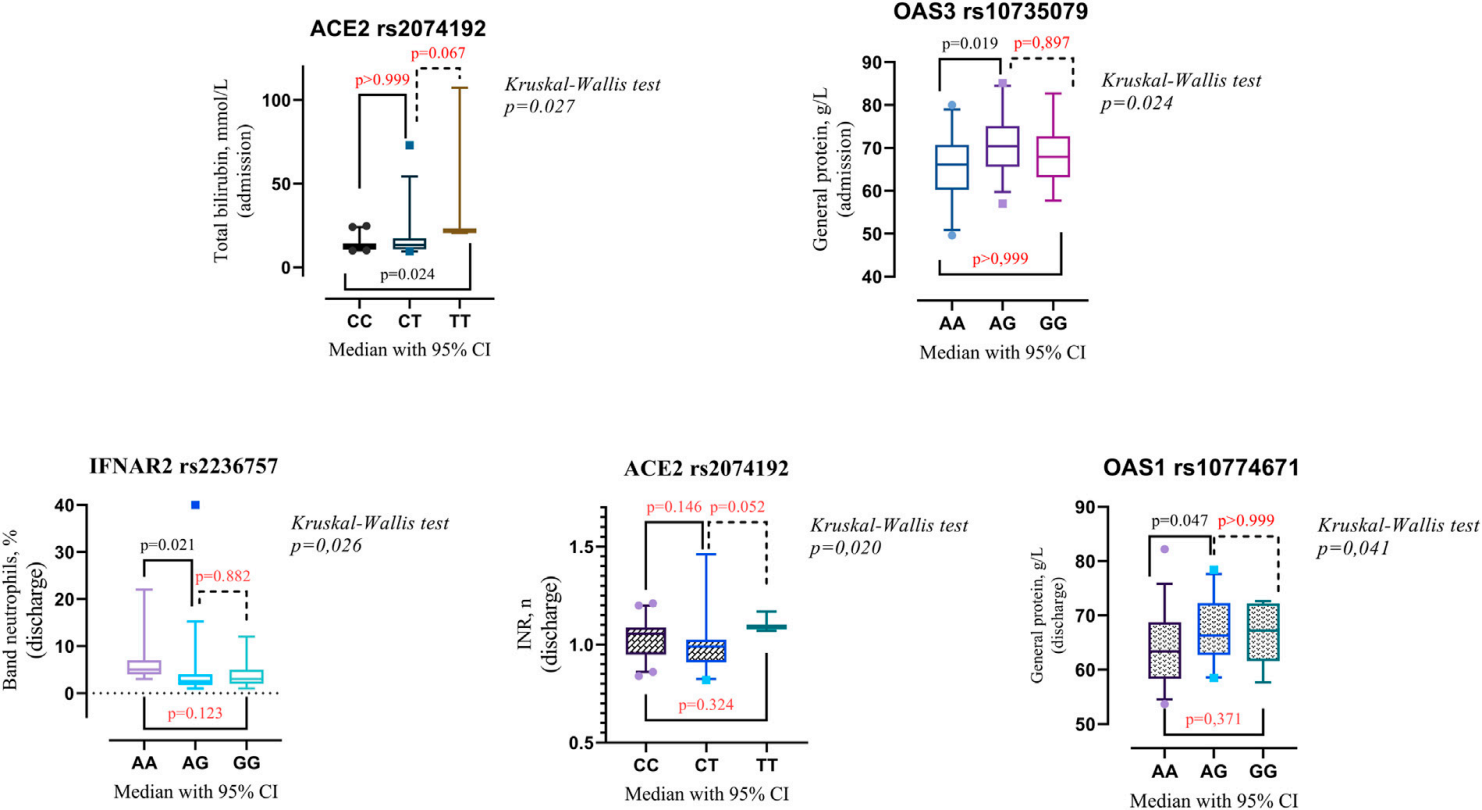
Comparison of the medians of laboratory findings in patients with different genotypes on admission/discharge: Band neutrophils, % (discharge) in patients with IFNAR2 rs2236757, total bilirubin g/L (admission) and INR, % (discharge) in patients with ACE2 rs2074192, general protein g/L (discharge) in patients with OAS1 rs10774671, general protein g/L (admission) in patients with OAS3 rs10735079. Kruskal–Wallis test with Dunn’s multiple comparisons test, as appropriate.

At discharge, patients with the IFNAR2 rs2236757 AA genotype displayed a significantly greater band neutrophil level than did those with the AG genotype (median 5 mmol/L, IQR 4–7 mmol/L vs. median 2.5 mmol/L, IQR 1.75–4 mmol/L, *p* = 0.021). Conversely, the OAS1 rs10774671 AA genotype was associated with a lower level (median 63.5 g/L, IQR 58.1–68.7 g/L vs. median 66.3 g/L, IQR 62–72.3 g/L, *p* = 0.047). Detailed data visualizations are presented in Figure 2 (medians with 95% CIs), while Table 7 provides the IQRs.

**TABLE 7:** Differences in clinical and laboratory findings at admission/discharge among patients with different genotypes.

Admission				
ACE2 rs2074192 genotype				
	СС (n = 40)	СT (n = 29)	TT (n = 3)	*p*-value^a^
Total bilirubin, mmol/L, IQR^b^	12.2 (10.7–14.2)	13.4 (10.8–17.4)	21.9 (20.5–107)	*p* = 0.027
OAS3 rs10735079 Genotype				
	AA (n = 30)	AG (n = 32)	GG (n = 10)	*p*-value^a^
General protein, g/L	66.2 (60.2–70.7)	70.4 (65.6–75.1)	68 (63.2–72.7)	*p* = 0.024
Discharge				
IFNAR2 rs2236757 Genotype				
	AA (n = 7)	AG (n = 34)	GG (n = 31)	*p*-value^a^
Band neutrophils, %	5 (4–7)	2.5 (1.75–4)	3 (2–5)	*p* = 0.026
ACE2 rs2074192 Genotype				
	СС (n = 40)	СT (n = 29)	TT (n = 3)	*p*-value^a^
INR, n	1.06 (0.95–1.09)	0.99 (0.91–1.03)	1.09 (1.07–1.17)	*p* = 0.020
OAS1 rs10774671 Genotype				
	AA (n = 32)	AG (n = 30)	GG (n = 10)	*p*-value^a^
General protein, g/L	63.5 (58.1–68.7)	66.3 (62–72.3)	67.3 (61.6–72.2)	*p* = 0.041

^a^ Kruskal–Wallis test as appropriate.

^b^ IQR, interquartile range.

The Kruskal‒Wallis test revealed a statistically significant difference in INR among patients categorized by their ACE2 rs2074192 genotype (*p* = 0.020). However, Dunn’s multiple comparisons test, employed to identify specific pairwise differences, did not detect any significant variations in INR between individual genotypes (Figure 2).

### 3.4 Correlation analysis

**FIGURE 3:**
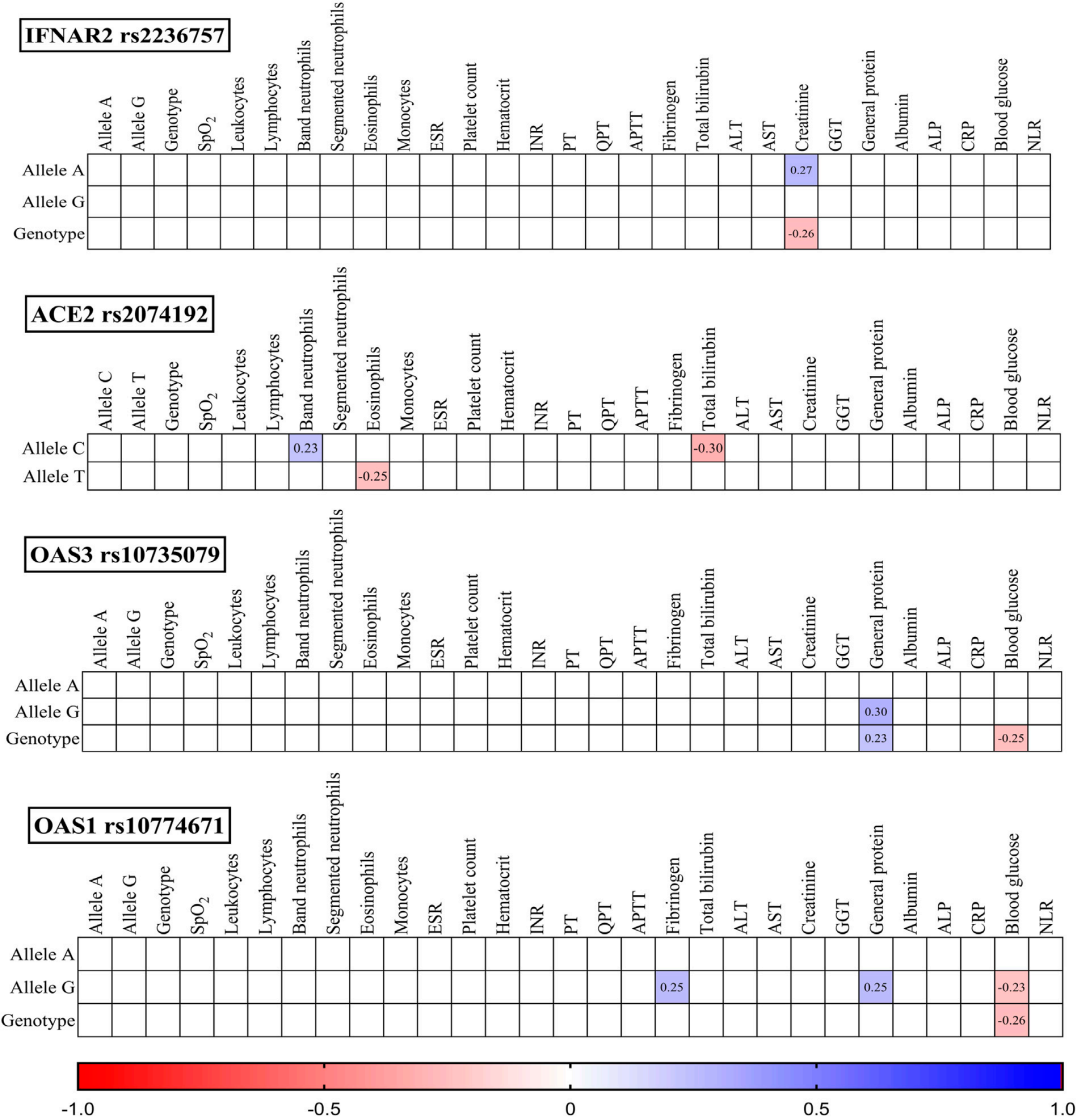
Correlation correlogram. Laboratory findings on admission (likely continuous and binary data based on the mention of point-biserial correlation). Point-biserial correlation: Used for correlations between binary (yes/no) data and continuous data. Red: Strong negative correlation (r = −1.0). Blue: Strong positive correlation (r = 1.0). Only statistically significant correlations (*p*-value ≤ 0.05) are shown.

**FIGURE 4:**
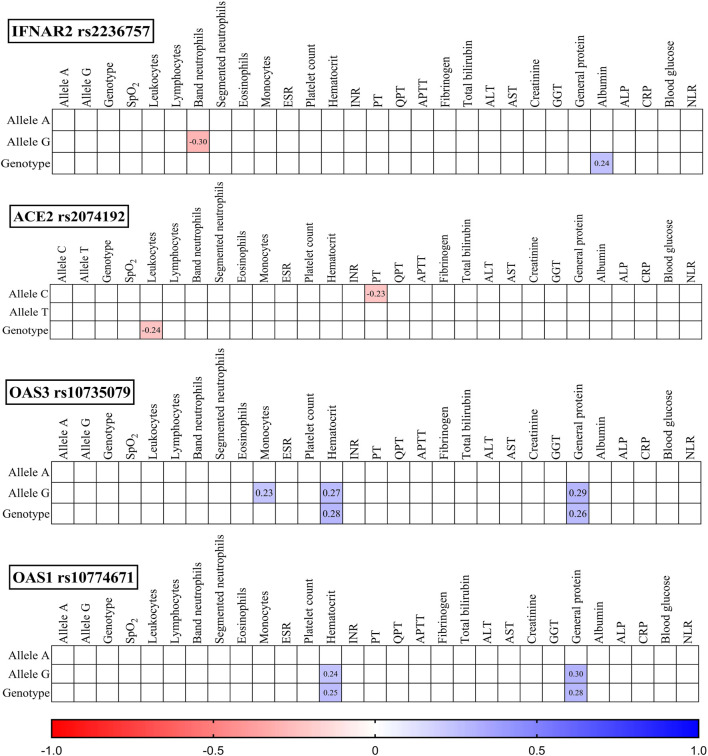
Correlation correlogram. Laboratory findings at discharge (likely continuous and binary data based on the mention of point-biserial correlation). Point-biserial correlation: Used for correlations between binary (yes/no) data and continuous data. Red: Strong negative correlation (r = −1.0). Blue: Strong positive correlation (r = 1.0). Only statistically significant correlations (*p*-value ≤ 0.05) are shown.

To investigate potential associations, we analysed the studied SNPs, their corresponding genotypes, and various laboratory outcomes measured upon admission (Figure 3) and discharge (Figure 4).

### 3.5 Admission findings

IFNAR2 rs2236757: The presence of the A allele was positively correlated with creatinine levels (r = 0.27, *p* = 0.021), while the GG genotype was negatively correlated with creatinine levels (r = −0.26, *p* = 0.027).

ACE2 rs2074192: The C allele displayed a positive correlation with band neutrophils (r = 0.23, *p* = 0.047) and a negative correlation with total bilirubin (r = −0.30, *p* = 0.010). Conversely, the T allele was negatively correlated with eosinophils (r = −0.25, *p* = 0.035).

OAS3 rs10735079: Both the G allele (r = 0.30, *p* = 0.011) and the GG genotype (r = 0.23, *p* = 0.047) were positively correlated with general protein levels. Additionally, the GG genotype was negatively correlated with blood glucose (r = −0.25, *p* = 0.036).

OAS1 rs10774671: The G allele was positively correlated with fibrinogen (r = 0.27, *p* = 0.033) and general protein (r = 0.25, *p* = 0.032) and negatively correlated with blood glucose (r = −0.23, *p* = 0.050). Similarly, the GG genotype exhibited a negative correlation with blood glucose (r = −0.26, *p* = 0.030).

### 3.6 Discharge findings

IFNAR2 rs2236757: The G allele was negatively correlated with the band neutrophil count (r = −0.30, *p* = 0.012), while the GG genotype was positively correlated with the serum ALB concentration (r = 0.24, *p* = 0.038).

ACE2 rs2074192: The TT genotype was negatively correlated with leukocytes (r = −0.24, *p* = 0.041), and the C allele was positively correlated with prothrombin time (PT) (r = −0.23, *p* = 0.048).

OAS3 rs10735079: The G allele was positively correlated with monocytes (r = 0.23, *p* = 0.048), haematocrit (r = 0.27, *p* = 0.023), and general protein (r = 0.29, *p* = 0.015). The GG genotype was also positively correlated with haematocrit (r = 0.28, *p* = 0.019) and general protein (r = 0.26, *p* = 0.025).

OAS1 rs10774671: Both the GG genotype and G allele were positively correlated with haematocrit (r = 0.25, *p* = 0.033 and r = 0.24, *p* = 0.043, respectively) and general protein (r = 0.28, *p* = 0.019 and r = 0.30, *p* = 0.010, respectively).

Notably, the correlations observed between the variables were statistically weak.

### 3.7 Regression analysis

We created a simple logistic regression model for predicting COVID-19 severity. This predictive model was developed based on the IFNAR2 rs2236757 allele G, ACE2 rs2074192 allele T, OAS1 rs10774671 allele G, SpO2 level (admission) and segmented neutrophil level on admission Table 8.

**TABLE 8:** Identification of risk factors for COVID-19 severity using logistic regression analysis.

	B (OR^a^)	S.E.	Wald	df	Sig. (*p*-value)	Exp(B)
IFNAR2 rs2236757 allele G	−1.679	1.683	0.995	1	0.318	0.186
ACE2 rs2074192 allele T	2.024	1.055	3.678	1	0.055	7.569
OAS1 rs10774671 allele G	2.437	1.252	3.789	1	0.052	11.442
SpO_2_ (admission)	−1.384	0.389	12.663	1	0.000	0.250
Segmented neutrophils (%, admission)	0.095	0.040	5.782	1	0.016	1.100
Constant	124.716	35.574	12.291	1	0.000	1.457E + 54

Variable(s) entered in step 1: IFNAR2 rs2236757 allele G, ACE2 rs2074192 allele T, OAS1 rs10774671 allele G, SpO_2_ (admission), and segmented neutrophils (%, admission); odds ratios.

The resulting logistic regression model demonstrated statistical significance (*p* < 0.001), indicating a strong association between the selected predictors and COVID-19 severity. Additionally, the Nagelkerke R-squared of 0.763 indicates that the independent variables in the model explain a substantial proportion (76.3%) of the variation in COVID-19 severity.

**FIGURE 5:**
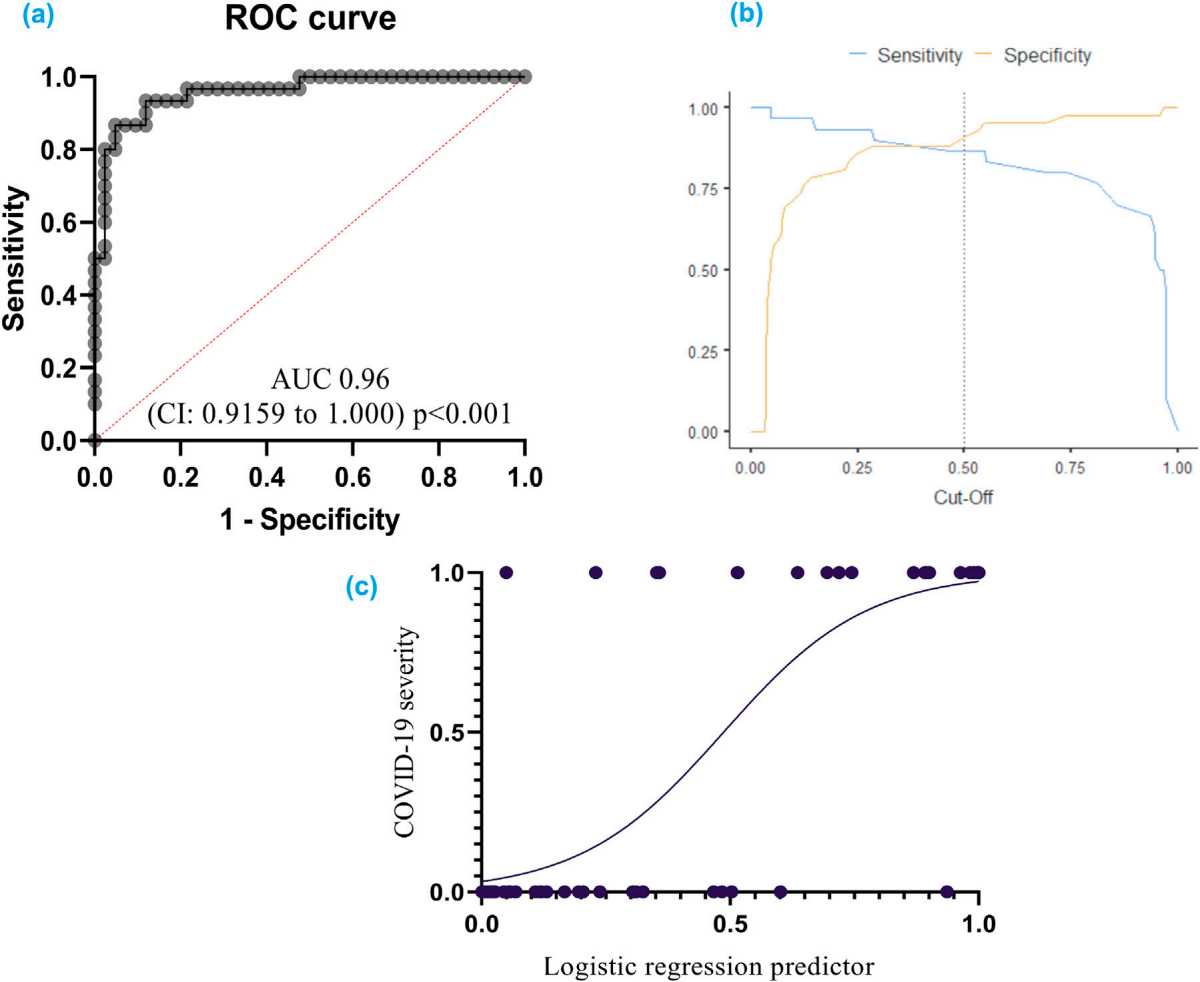
**(A)** ROC curve for COVID-19 severity: This curve shows how well the model can distinguish between different severities of COVID-19; **(B)** Cut-off plot for optimal prediction: This plot helps determine the best threshold for the model’s predictions; **(C)** Simple logistic regression curve – a statistical method employed to predict a binary outcome based on a single predictor variable. Additionally, it quantifies the numerical relationship between these two variables. A receiver operating characteristic (ROC) curve graphically represents the performance of a classification model across various classification thresholds. The area under the ROC curve (AUC) serves as a comprehensive metric for evaluating the model’s ability to discriminate between positive and negative outcomes across all potential cut-off points.

The developed model achieved a high level of accuracy (90.3%), signifying its ability to accurately classify cases into severity categories. Receiver operating characteristic (ROC) analysis confirmed the model’s effectiveness. The ROC curve analysis yielded an area under the curve (AUC) of 0.96 (95% CI: 0.92–1.00), demonstrating excellent ability to discriminate between patients with severe and nonsevere COVID-19 (Figure 5).

The model exhibited a specificity of 92.9% and a sensitivity of 86.7%. The specificity refers to the model’s ability to correctly identify individuals without severe COVID-19, while the sensitivity reflects its accuracy in detecting severe cases.

The cut-off value for logistic function (p) was established at 0.5. This threshold can be used to categorize patients into high-risk and low-risk groups based on the probability of having severe COVID-19.

### 3.8 Genmania interactione

**FIGURE 6:**
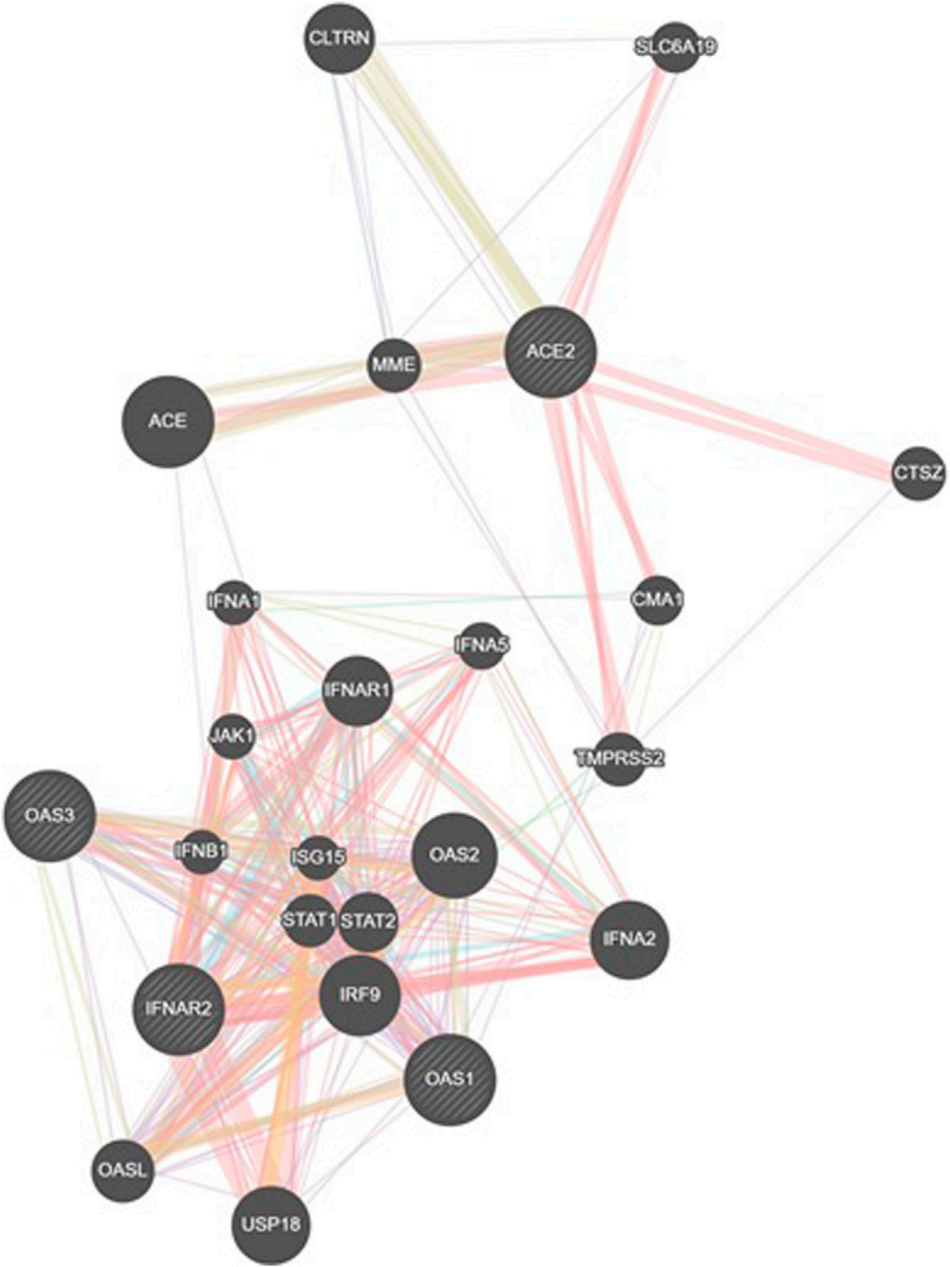
GeneMANIA network analysis was employed to visualize the interaction network between the studied genes (IFNAR2, ACE2, OAS1, OAS3) and other relevant genes. This figure illustrates gene interactions determined by physical interactions, co-expression patterns, predicted associations, co-localization, genetic relationships, shared biological pathways, and common protein domains.

An analysis using GeneMANIA network data (GENEMANIA, 2024) revealed interconnections between the investigated SNPs and other polymorphisms, including those of CLTRN (collectrin, an amino acid transport regulator), SLC6A19 (solute carrier family 6 member 19), MME (membrane metalloendopeptidase), ACE (angiotensin I converting enzyme), CTSZ (cathepsin Z), CMA1 (chymase 1), IFNA1 (interferon alpha 1), IFNA5 (interferon alpha 5), IFNAR1 (interferon alpha and beta receptor subunit 1), JAK1 (janus kinase 1), TMPRSS2 (transmembrane serine protease 2), IFNB1 (interferon beta 1), ISG15 (ubiquitin like modifier), STAT1 (signal transducer and activator of transcription 1), STAT2 (signal transducer and activator of transcription 2), IRF9 (interferon regulatory factor 9), OASL (2′-5′-oligoadenylate synthetase like), USP18 (ubiquitin specific peptidase 18), IFNA2 (interferon alpha 2), and OAS2 (2′-5′-oligoadenylate synthetase 2) (Figure 6).

The analysis revealed that physical interactions between genes in the proposed network were documented in a high percentage of cases (77.64%), coexpression was found in a smaller proportion (8.01%), and the predicted functional relationships between genes were 5.37%, colocalization was 3.63%, genetic interactions were 2.87%, pathways were 1.88% and shared protein domains were 0.60% Table 9.

**TABLE 9:** GenMANIA interaction.

Physical interactions	Coexpression	Predicted	Colocalization	Genetic interactions	Pathway	Shared protein domains
ACE–ACE2	OAS2–OAS1	OAS2–OAS3	IFNB1–IFNAR2	TMPRSS2–OASL	IRF9–IFNAR2	OAS1–OAS3
IRF9–OAS3	IRF9–OAS1	OASL–OAS1	OAS1–OAS3	TMPRSS2–STAT2	IFNA2–IFNAR2	ACE–ACE2
IRF9–OAS1	OASL–OAS1	JAK1–IFNAR2	STAT2–OAS3	JAK1–STAT2	IFNAR1–IFNAR2	OAS2–OAS3
IRF9–IFNAR2	STAT1–OAS1	IRF9–IFNAR2	STAT2–OAS1	IFNA1–CMA1	STAT2–IFNAR2	OAS2–OAS1
IFNA2–FNAR2	OAS1–OAS3	STAT1–IFNAR2	STAT1–OAS3		STAT1–IFNAR2	CLTRN–ACE2
USP18–IFNAR2	ISG15–OAS1				IFNA5–IFNAR2	IFNAR1–IFNAR2
IFNAR1–IFNAR2	USP18–OAS1				IFNB1–IFNAR2	OASL–OAS3
STAT2–OAS3	ACE–ACE2				IFNAR1–IFNAR2	OASL–OAS1
STAT2–OAS1	OAS1–OAS3					
STAT2–IFNAR2	OAS2–OAS3					
CTSZ–ACE2	USP18–OAS1					
MME–ACE2	CLTRN–ACE2					
STAT1–OAS3	OASL–OAS3					
STAT1–IFNAR2	MME–ACE2					
CMA1–ACE2	STAT1–OAS3					
IFNA5–IFNAR2	ISG15–OAS3					
JAK1–IFNAR2	STAT2–ACE					
IFNA1–IFNAR2	IFNAR1–IFNAR2					
IFNB1–IFNAR2	STAT1–IFNAR2					
ACE–ACE2	CLTRN–ACE2					
TMPRSS2–ACE2	USP18–OAS3					
SLC6A19–ACE2	MME–ACE2					
	SLC6A19–ACE2					
	CLTRN–ACE2					
	TMPRSS2–OAS1					
	CMA1–OAS1					
	IFNB1–IFNAR2					
	STAT2–OAS1					

## 4 Discussion

This study investigated the potential influence of SNPs in genes associated with the interferon pathway (IFNAR2 rs2236757), antiviral response (OAS1 rs10774671, OAS3 rs10735079), and viral entry (ACE2 rs2074192) on COVID-19 severity and their association with MAFLD.

Patients with MAFLD have increased inflammatory mediator levels (Duan et al., 2022). Previous research linked the interferon pathway to MAFLD pathogenesis (Møhlenberg et al., 2019) and COVID-19 severity (Bastard et al., 2021).

Several lines of investigation, including genome-wide association studies (GWASs), transcriptomic analyses, and single-cell studies, have focused on IFNAR2 (rs2236757) as a potential genetic risk factor for severe COVID-19 (Pairo-Castineira et al., 2020). Supporting evidence has emerged from studies focused on genes associated with COVID-19 severity, where a specific variant within the IFNAR2 gene (rs2236757) has been linked to poorer clinical outcomes (Pairo-Castineira et al., 2020; Fricke-Galindo et al., 2022). Notably, this variant (rs2236757) has been associated with an increased risk of critical illness and mortality from COVID-19 infection (Fricke-Galindo et al., 2022). Interestingly, research suggests an inverse correlation between soluble IFNAR2 protein (sIFNAR2) levels and COVID-19 outcomes, with lower levels observed in deceased patients and higher levels in survivors (Fricke-Galindo et al., 2022).

Dieter et al. (2022) further strengthened the connection between the IFNAR2 rs2236757 genotype and severe COVID-19 by demonstrating an association with increased ICU admission rates and mortality (Dieter et al., 2022). However, our own investigation did not reveal a significant association between the rs2236757 polymorphism and COVID-19 severity in our patient population. Additionally, this SNP did not exhibit a relationship with MAFLD.

Although our findings did not reveal a substantial impact of the IFNAR2 rs2236757 genotype on COVID-19 severity or clinical outcomes, an interesting observation emerged. Patients with the A allele of rs2236757 presented with higher creatinine levels upon admission than did those without the A allele. Conversely, upon discharge, patients with the G allele displayed a negative correlation with band neutrophils, potentially indicating lower neutrophil counts. Moreover, the distribution of the IFNAR2 rs2236757 genotype did not significantly differ between MAFLD patients and non-MAFLD patients in our study.

2′–5′ oligoadenylate synthetases (OASs) are a family of interferon-stimulated antiviral enzymes crucial for the innate immune response (Kristiansen et al., 2011). These enzymes recognize viral double-stranded RNA (dsRNA) and trigger RNA destabilization via RNase L activation within infected cells. Humans possess four OAS genes (OAS1, OAS2, OAS3, and OASL) located on chromosomes 12q24.1 (OAS1-3) and 12q24.2 (OASL). OAS1, a dsRNA-activated enzyme, plays a central role in cellular antiviral defense (Fagone et al., 2016). OAS3 encodes a single transcript that results in the activation of latent RNase L, leading to the degradation of both viral and cellular RNA (Fagone et al., 2016).

Previous studies have explored the potential association between OAS polymorphisms and COVID-19 severity. Banday et al. (2022) investigated the influence of OAS1 rs10774671 on COVID-19 hospitalization outcomes and suggested that it has a functional impact on disease severity (Banday et al., 2022). Similarly, the GenOMICC study by Pairo-Castineira et al. identified a potential role for OAS3 rs10735079 in the progression to critical illness in COVID-19 patients (Pairo-Castineira et al., 2020).

Our study population’s genotype and allele distribution for the investigated SNPs (rs10774671 and rs10735079) did not differ significantly from those of the European population. Furthermore, we did not observe statistically significant differences in COVID-19 severity between patients with either OAS1 rs10774671 or OAS3 rs10735079. Interestingly, carrying two copies of the G allele (homozygous genotype) for both polymorphisms was linked to variations in certain clinical parameters. In rs10774671 carriers, the G allele correlated with increased protein and haematocrit levels, as well as increased fibrinogen. Patients with rs10735079 and the G allele exhibited increased monocyte levels. Importantly, no significant differences in the distribution of these polymorphisms were found between patients with and without MAFLD. The G allele OAS1 rs10774671 was used in logistic regression as a predictor of COVID-19 severity (*p* = 0.052).

2′–5′ oligoadenylate synthetases (OASs) are interferon-induced antiviral enzymes that recognize virally produced dsRNA and initiate RNA destabilization through the activation of RNase L within infected cells (Kristiansen et al., 2011). The human genome encodes four 2′,5′-oligoadenylate synthetase (OAS) genes: OAS1, OAS2, OAS3, and OASL. These genes map to distinct chromosomal locations, with OAS1-3 clustered on chromosome 12q24.1 and OASL situated on chromosome 12q24.2. Notably, OAS1 functions as a dsRNA-activated antiviral enzyme, playing a crucial role in the innate immune response against viral infection. OAS3 encodes a single transcript that produces an enzyme that activates latent RNase L, leading to the degradation of both viral and endogenous RNA (Fagone et al., 2016).

Banday et al. (2022) showed the influence of OAS1 rs10774671 on hospitalization outcomes for COVID-19 patients and proposed a functional impact on COVID-19 severity (Banday et al., 2022). In the GenOMICC study, Pairo-Castineira et al. reported that OAS3 rs10735079 plays a role in the progression of critical illness in COVID-19 patients (Pairo-Castineira et al., 2020). The studied population did not differ from the European population in terms of SNP genotype or alleles. No statistically significant differences in COVID-19 severity were found between patients with OAS1 rs10774671 and patients with OAS3 rs10735079 in our study. The presence of the G allele was associated with increased protein and haematocrit levels, increased fibrinogen levels in rs10774671 patients and increased monocyte levels in rs10735079 patients. When comparing patients with MAFLD and non-MAFLD, no difference in the distribution of these polymorphisms was found.

The ACE2 receptor and transmembrane serine protease 2 (TMPRSS2) are established factors facilitating SARS-CoV-2 entry into human cells (Zipeto et al., 2020). Viral entry begins with the binding of the viral spike (S) protein to ACE2, followed by S protein cleavage mediated by TMPRSS2. This cleavage allows for the fusion of the viral membrane with the host cell membrane, enabling viral replication and spread within the target cells (Zipeto et al., 2020). Genetic polymorphisms that influence the expression of these genes could impact the course of SARS-CoV-2 infection (Sheikhian et al., 2023). Studies have shown an association between the ACE2 rs2074192 TT genotype and increased COVID-19 mortality (Sheikhian et al., 2023).

Furthermore, the severity of COVID-19 has been linked to preexisting conditions such as cardiovascular disease, retinopathy in hypertensive individuals, type 2 diabetes, and hypertensive left ventricular hypertrophy, which are more prevalent in carriers of the T allele of the rs2074192 polymorphism (Liu et al., 2018; Fan et al., 2019). These conditions, including MAFLD, are often manifestations of metabolic syndrome.

Our investigation focused on MAFLD and did not reveal any significant differences in the distribution of ACE2 rs2074192 genotypes or alleles between the MAFLD and non-MAFLD groups. Additionally, the presence of the ACE2 rs2074192 polymorphism did not seem to influence COVID-19 severity in our study population. It is important to note that our population exhibited a distinct distribution of genotypes and alleles compared to the European reference population.

Our findings do not support a significant association between the investigated genes (IFNAR2 rs2236757, ACE2 rs2074192, OAS1 rs10774671, and OAS3 rs10735079) and the course of SARS-CoV-2 infection. Furthermore, we did not observe an increased prevalence of these polymorphisms in patients with MAFLD. Additionally, no interaction was identified between these polymorphisms and MAFLD that could worsen the course of COVID-19 in this patient population.

To further explore the potential interconnections among the investigated SNPs and other relevant genes, we conducted a GeneMANIA network analysis. This analysis revealed a complex network involving the studied SNPs and additional genes implicated in various biological processes, including immune response, inflammation, and viral infection. Notably, a high proportion of identified interactions were based on documented physical interactions between gene products, suggesting a potential for direct protein-protein interactions.

Despite the absence of significant associations observed in this study between the SNPs under investigation and COVID-19 severity, the possibility that the study was underpowered to detect such associations warrants further exploration.

Based on these findings, the following clinical implications can be discussed.

The absence of a significant association between the studied SNPs and COVID-19 severity may provide some reassurance that these genetic variants do not play a major role in determining disease outcome. However, it is essential to acknowledge the limitations of the study and emphasize the need for further research to explore the potential role of other genetic factors.

The observed associations between IFNAR2, OAS1, and OAS3 with specific laboratory parameters (creatinine, protein, hematocrit, monocytes) warrant further investigation. These findings could potentially identify novel biomarkers for disease progression or severity. To understand the underlying mechanisms, future studies could explore the functional impact of these SNPs on gene expression and protein function.

The lack of association between MAFLD and the studied SNPs suggests that these genetic factors do not exacerbate the risk of severe COVID-19 in patients with MAFLD. This finding emphasizes the importance of focusing on other factors, such as metabolic dysregulation, inflammation, and immune response, in understanding the interplay between MAFLD and COVID-19.

The observed differences in SNP distribution between the study population and the European population highlight the importance of considering population-specific factors in genetic studies.

Furthermore, the severity of COVID-19 has been linked to preexisting conditions such as cardiovascular disease, retinopathy in hypertensive individuals, type 2 diabetes, and hypertensive left ventricular hypertrophy, which are more prevalent in carriers of the T allele of the rs2074192 polymorphism (You et al., 2020; Møhlenberg et al., 2019). These conditions, including MAFLD, are often manifestations of metabolic syndrome.

## 5 Limitation

The present study is subject to several limitations. Firstly, the sample size, comprising 96 participants distributed across three groups, may be insufficient to identify subtle genetic associations, potentially limiting the study’s statistical power. Secondly, the population-specific nature of the investigation, focusing exclusively on individuals of Ukrainian ethnicity, restricts the generalizability of the findings to other populations. Thirdly, the monocentric design of this study inherently restricts the generalizability of the findings due to a limited population sample size, thereby increasing the potential for selection bias.

We acknowledge the potential for confounding factors to influence the observed relationship between the studied SNPs and COVID-19 severity. To address this, we adjusted for age, sex, body mass index, comorbidities (including hypertension, diabetes, and cardiovascular disease) in our analyses. While these adjustments mitigate the impact of known confounders, the possibility of residual confounding cannot be entirely excluded. Further studies with additional covariates or alternative study designs may be necessary to refine our understanding of the relationship between these genetic variants and COVID-19 severity.

## 6 Conclusion

This study contributes to the ongoing investigation of genetic factors influencing COVID-19 severity. We did not identify a strong association between the studied SNPs and disease severity. The distinct distribution of ACE2 rs2074192 genotypes in our population compared to the reference group suggests the need for further research considering ethnicity and geographic factors. Future studies could explore functional analyses of these polymorphisms and investigate interactions with other genetic and environmental variables to gain a more comprehensive understanding of the complex interplay of factors influencing COVID-19 outcomes.

## Data Availability

The original contributions presented in the study are included in the article/supplementary material, further inquiries can be directed to the corresponding authors.
